# Supplementary material for: Gut bacterial dysbiosis and instability is associated with the onset of complications and mortality in COVID-19
Source: Gut Microbes. 2022 Feb 17;14(1):2031840. doi: 10.1080/19490976.2022.2031840 (PMC8855857; doi:10.1080/19490976.2022.2031840)
Supplement: Supplemental Material [file KGMI_A_2031840_SM4528.zip › supplementary/SupplTable1.docx]

| **Group** | **Antibiotics** | **Spectrum of activity** | **Special features** |
| --- | --- | --- | --- |
| **1 = Broad-spectrum** | **- Beta-lactam antibiotics** (Acylaminopenicillins, Aminopenicillins, Carbapenems)  **- Tetracyclins**  **- Levofloxacin**  **- Moxifloxacin** | - Anaerobic bacteria  - Gram-positive bacteria  - Gram-negative bacteria |  |
| **2 = Narrow-spectrum** | - **Linezolid**  **- Daptomycin**  **- Flucloxacillin**  **- i.v. Vancomycin** | - Gram-positive bacteria |  |
| **3 = Cephalosporins** | **- Cephalosporins** | - Anaerobic bacteria  (partly)  - Gram-positive bacteria  - Gram-negative bacteria | - lack of activity against enterococci |
| **4 = Others** | **- Gentamicin**  **- Ciprofloxacin**  **- Azithromycin** | - Gram-positive bacteria (partly for Aminoglycosides),  - Gram-negative bacteria |  |
